# Diarrhoea and smoking: an analysis of decades of observational data from Bangladesh

**DOI:** 10.1186/s12889-015-1906-z

**Published:** 2015-07-12

**Authors:** Sumon Kumar Das, Mohammod Jobayer Chisti, A.M. Shamsir Ahmed, Mohammad Abdul Malek, Shahnawaz Ahmed, K.M. Shahunja, Farzana Ferdous, Fahmida Dil Farzana, Jui Das, Aminur Rahman, Abdullah Al Mamun, Abu Syed Golam Faruque

**Affiliations:** Centre for Nutrition and Food Security (CNFS), International Centre for Diarrhoeal Disease Research, Dhaka, Bangladesh; School of Public Health, The University of Queensland, Brisbane, Australia; Department of Clinical Trial and Clinical Epidemiology, Graduate School of Comprehensive Human Sciences, University of Tsukuba, Ibaraki, Japan

**Keywords:** Bangladesh, Diarrhoea, Smoking, Urban areas

## Abstract

**Background:**

Although cigarette smoking affects all biological systems of the human body including the gastrointestinal tract, there is a lack of evidence regarding its effect on the severity of diarrhoeal disease and whether a dose–response relationship exists. We therefore tested for the presence of specific causative pathogens for infectious diarrhoea, assessed the independent effect of smoking on its severity and tested whether any dose–response relationship existed while controlling for subjects’ age, sociodemographic characteristics and presence of causative pathogens in an urban setting in Bangladesh.

**Methods:**

A total of 20,757 patients aged 15 years and above with diarrhoea were enrolled into the Diarrhoeal Disease Surveillance System, managed by the International Centre for Diarrhoeal Disease Research, Bangladesh, from 1993 to 2012. We collected data on individuals’ current daily consumption of cigarettes and bidis (traditional hand-rolled cigarettes) and conducted an ordered logistic regression to determine the effect of smoking on diarrhoeal disease severity and whether a dose–response relationship exists.

**Results:**

We identified 19 % of patients with diarrhoea as smokers, of whom 52 % smoked 1–9 cigarettes per day. While 97 % of smokers were male, 41 % were aged 15–30 years of age. Smokers were found to have a significantly lower severity of diarrhoeal disease (OR: 0.92, 95 % CI: 0.85–0.99, p = 0.025) after adjusting for age, wealth quintile, illiteracy and the presence of specific causative pathogens (*Vibrio cholerae* and *Shigella*). We observed no dose–response relationship between the number of cigarettes smoked per day and disease severity when adjusting for the same covariates. Smokers were more frequently infected with *Shigella* (7 vs. 6 %, p < 0.001) and less often with *Vibrio cholerae* (22 vs. 26 %, p < 0.001) than their non-smoking counterparts.

**Conclusions:**

The aetiology and severity of diarrhoeal disease differed between smokers and non-smokers in our sample. However, we found no dose–response relationship between disease severity and the number of cigarettes smoked per day.

## Background

Despite increasing awareness of the health harms of smoking, tobacco use remains highly prevalent in low income countries [1–9]. The World Health Organization estimates that one third of the global population aged 15 years and above are smokers and that 84 % of these live in developing countries [2, 3]. Cigarettes contain nicotine, which is known to have a number of detrimental effects on a range of biological systems including the respiratory, cardiovascular, gastrointestinal, renal nervous, endocrine and metabolic systems [4–8]. Furthermore, previous work suggests that a dose–response relationship exists between cigarette smoking and the severity of chronic conditions such as ulcerative colitis, gastroesophageal reflux disorder, peptic ulcer disease and Crohn’s disease, in addition to the risk of developing malignant tumours [8, 9]. Similar relationships have also been documented with regards to exposure to second-hand tobacco smoke [10-13].

Although diarrhoea represents the second leading cause of childhood morbidity and mortality globally, it also often affects other age groups including adults and older people [14]. *Vibrio cholerae* and *Shigella*, both of which have high pandemic potential, are the two most important causative agents for diarrhoeal disease and are considered major public health concerns in a number of countries [15–20]. The majority of studies on the epidemiology of diarrhoeal disease have focused on patients’ age, the etiology and burden of disease, and specific outcomes, including mortality [15]. However, there is currently a lack of evidence on individual-level determinants of diarrhoeal disease, in particular health behaviours such as smoking. The International Centre for Diarrhoeal Disease Research, Bangladesh (icddr,b) has maintained a comprehensive Diarrhoeal Disease Surveillance System (DDSS) since 1979 [21], which collects prospective data on individuals presenting with diarrhoea to a large urban health centre in Dhaka. Data on smoking behavior were recorded for all attending individuals aged 15 years and above as part of this surveillance system. Using this data, the aim of the present study were firstly to assess the distributions of causative pathogens for diarrhoeal disease among smokers and non-smokers, secondly, to assess the symptom profiles of infectious diarrhoea cases among smokers and non-smokers when stratified by the presence of different causative pathogens, and, thirdly, to determine the independent effects of smoking on disease severity while controlling for patients’ age, sociodemographic characteristics and the presence of specific causative pathogens and to test for the presence of a dose–response relationship.

## Methods

### Study context and data collection

Dhaka Hospital, located in the capital city of Bangladesh, was established in 1962 by icddr,b and has since provided free medical care to all patients. The DDSS systematically sampled 4 % of all attending patients from 1979 to 1995 and 2 % of patients since 1996 to adjust for a more than two-fold increase in patient numbers. Using a structured questionnaire, the system collects information on infant and young children’s feeding practices and the use of drug and fluid therapy in the home, as well as patients’ clinical, epidemiological, etiologic, and demographic characteristics. We extracted the relevant data, which covered the period from 1993 to 2012, from the DDSS archive. A total of 20,914 patients with diarrhoea aged 15 years and above were enrolled into the surveillance system during this period. Of these, 20,757 were included in the analysis and 157 were excluded because of missing data.

### Assessment of smoking status

As part of DDSS, current use of cigarettes or bidis (traditional hand-rolled cigarettes) was recorded for each enrolled patient aged 15 years and above with diarrhoea, in addition to the number smoked per day. Although data on parental smoking behavior was collected for children under 15 years, this was not used in the present analysis. Current smokers (number of cigarettes or bidis smoked per day ≥1) were considered cases while non-smokers (number smoked per day = 0) comprised the comparison group.

### Laboratory methods

A fresh whole stool specimen was collected from each patient enrolled in the DDSS and examined in icddr,b’s central laboratory in Dhaka. Each specimen was aliquoted into three containers and submitted for routine screening for common enteric pathogens including *Vibrio cholerae, Shigella* spp., *Salmonella* spp., *Campylobacter* spp., *Entamoeba histolytica*, *Giardia lamblia* [22], and rotavirus [23] using standard laboratory methods.

The antimicrobial susceptibility of *Shigella* spp. and *Vibrio cholerae* to different antimicrobial agents was determined using the disk diffusion method (CLSI 2010) employing commercial antimicrobial discs (Oxoid, Basingstoke, United Kingdom). While we used ampicillin (10 μg), mecillinum (25 μg), nalidixic acid (30 μg), trimethoprim-sulfamethoxazole [(TMP-SXT)], (25 μg), and ciprofloxacin (5 μg) antibiotic discs to test the susceptibility of *Shigella,* tetracycline (30 μg), (TMP-SXT) (25 μg), erythromycin (15 μg), and ciprofloxacin (5 μg) disks were used for *Vibrio cholerae* [24].

### Data analysis

Patients’ sociodemographic and clinical characteristics, including severity of diarrhoeal disease symptoms (mild, moderately severe, severe or very severe), abdominal pain, stool character (watery), length of hospitalisation (>24 h) and distribution of enteric pathogens were compared between smokers and non-smokers using the chi-square test.

Diarrhoeal disease severity was scored using a 17-point numerical scale based on the following clinical features: duration of diarrhoea, number of stools passed in last 24 h, number of occasions of vomiting in last 24 h, fever (°C), dehydration status and treatment received (described in greater detail by Ruuska et.al.) [25]. Disease severity was then classified as mild (≤6), moderately severe (7–9), severe (10–12) or extremely severe (≥13). Duration of vomiting was not used for scoring because of incomplete data.

Wealth quintiles were estimated using principal component analysis based on household assets such as construction materials of the house and ownership of durable goods including a fan, radio, television, cupboard, sanitary toilet and a luxury or ordinary cot. Age was expressed as a binary variable, with subjects categorised being 15–30 years or over 30 years respectively. The annual US$ inflation rate was used when estimating the family income. Other binary variables were included to code for whether subjects habitually boiled their drinking water and whether they had taken antimicrobials before attending hospital.

Finally, we used an ordered logistic regression model (proportional odds model) to assess the independent effects of smoking on disease severity while controlling for age and sociodemographic factors (wealth index and level of education) and the presence of causative pathogens (*Shigella*, and *Vibrio cholerae*) using STATA Version 12. We performed an additional analysis by including a categorical variable for number of cigarettes smoked daily (1 = 1–9, 2 = 10–19, 3 = ≥20) to determine any dose–response relationship with diarrhoeal disease severity while controlling for the same covariates. Given that 97 % of smokers were male, female subjects were excluded from all multivariate analyses. The strengths of association were determined by estimating odds ratios (OR) and 95 % confidence intervals (95 % CI) with p <0.05 considered to be statistically significant.

### Ethical statement

The DDSS of icddr,b is an ongoing programme of the Dhaka Hospital which has been approved by the Research Review Committee and the Ethical Review Committee of icddr,b. Interviews took place only after obtaining verbal consent from either patients themselves, or, where patients were aged 15 to 19 years, from both patients and their parents or guardians, according to hospital policy. The questionnaire recorded when consent was given. Patients were assured of the confidentiality of all personal data collected from them and informed about its use for research purposes and for improving patient care. The Ethical Review Committee approved this method for obtaining consent and is satisfied that patients participated voluntarily, that their rights were not violated, and that personal data were handled in a confidential manner by the hospital staff.

## Results

We found that 19 % (3986) of patients diagnosed with diarrhoea were current smokers, 52 % of whom (2095) smoked 1–9 cigarettes per day, 37 % (1464) 10–19 cigarettes per day, and 11 % (427) 20 or more cigarettes per day (data not shown). The vast majority of smokers were male (97 %), 41 % were aged 15–30 years. A significantly higher proportion of smokers were illiterate, had a monthly household income of ≤100 US$ and were generally more socioeconomically disadvantaged when compared with non-smokers (Table 1). Furthermore, while a lower proportion of smokers boiled their drinking water, the proportion using antimicrobials before attending hospital was similar in both groups.

**Table 1:** Socioeconomic characteristics of diarrhoea patients identified as smokers and non-smokers

Indicators	Smoker, n = 3986 (%)	Non-smoker, n = 16,771 (%)	OR (95 % CI) *p* value
15–30 years	1630 (41)	9566 (57)	0.52 (0.49, 0.56) <0.001
Above 30 years	2356 (59)	7205 (43)	1.92 (1.79, 2.06) <0.001
Male sex	3874 (97)	7767 (46)	40.10 (33.02, 48.74) <0.001
Monthly income ≤100 US$	2752 (69)	10,664 (64)	1.28 (1.19, 1.38) <0.001
Small family size (≤5 mean)	2298 (58)	10,268 (61)	0.86 (0.80, 0.93) <0.001
Wealth quintile			
Rich	591 (15)	3557 (21)	0.65 (0.59, 0.71) <0.001
Upper middle	720 (18)	3417 (20)	0.86 (0.79, 0.94) <0.001
Middle	799 (20)	3317 (20)	1.02 (0.93, 1.11) 0.720
Lower middle	918 (23)	3307 (20)	1.22 (1.12, 1.32) <0.001
Poor	958 (24)	3173 (19)	1.36 (1.25, 1.47) <0.001
Patient’s illiteracy	1897 (52)	7328 (44)	1.17 (1.09, 1.25) <0.001
Boils drinking water	635 (16)	4019 (24)	0.60 (0.55, 0.66) <0.001
Administered antimicrobial therapy before attending the hospital	2258 (57)	9646 (58)	0.97 (0.90, 1.04) 0.328

*OR*, Odds ratio; *95 % CI*, 95 % Confidence interval. *p* values were calculated using the chi-square test

**Fig. 1:**
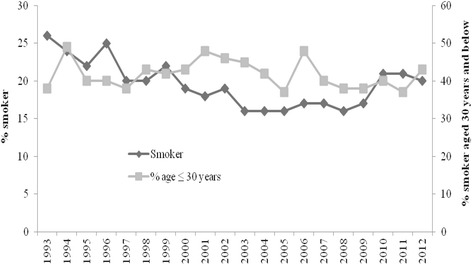
Yearly distribution of overall smokers and proportion of teenager and young adults smokers (1993–2012)

The overall proportion of smokers in 1993 was 26 %, which gradually decreased to 16 % in 2003 and thereafter began to increase steadily (Fig. 1). The proportion of teenage and young adult smokers also evolved over time and began to decrease gradually from 2005 except for a small rise in 2006 (Fig. 1).

Smokers were more frequently infected with *Shigella* spp. and less often with *Vibrio cholerae* than their non-smoking counterparts. The isolation rates of other common pathogens such as *Salmonella* spp., rotavirus, *Entamoeba histolytica*, and *Giardia lamblia* were similar in both groups (Table 2).

**Table 2:** Distribution of common causative pathogens for diarrhoea among smokers and non-smokers

Pathogens	Smoker, n = 3986 (%)	Non-smoker, n = 16,771 (%)	OR (95 % CI) *p* value
*Vibrio cholerae*	861 (22)	4409 (26)	0.77 (0.71, 0.84) <0.001
*Shigella* spp.	287 (7)	971 (6)	1.26 (1.10, 1.45) <0.001
*Salmonella* spp.	99 (3)	343 (2)	1.22 (0.97, 1.54) 0.096
*Campylobacter* spp.	186 (5)	814 (5)	0.96 (0.81, 1.13) 0.649
Rotavirus	130 (3)	612 (4)	0.90 (0.73, 1.09) 0.281
*Entamoeba histolytica*	81 (2)	278 (2)	1.24 (0.96, 1.60) 0.100
*Giardia lamblia*	97 (3)	364 (2)	1.13 (0.89, 1.43) 0.318

*OR*, Odds ratio; *95 % CI*, 95 % Confidence interval. *p* values were calculated using the chi-square test

In terms of disease severity, a significantly higher proportion of smokers had mild or moderate disease symptoms. A higher proportion of non-smokers, however, had severe symptoms (Table 3). The proportions of patients with watery stool and of those hospitalised for more than 24 h were significantly lower among smokers. The proportions of abdominal pain was similar in both groups (Table 3). Detailed pathogen-specific differences in disease severity and other clinical characteristics between smokers and non-smokers are also shown in Table 3.

**Table 3:** Disease severity and symptoms stratified by causative pathogen among smokers and non-smokers

Indicators	Overall n (%)		*Vibrio cholerae* n (%)		*Shigella* spp. n (%)		*Salmonella* spp. n (%)		*Campylobacter* spp. n (%)		Rotavirus n (%)		*Entamoeba histolytica n* (%)		*Giardia lamblia* n (%)	
	Sm;	Non-sm;	Sm;	Non-sm;	Sm;	Non-sm;	Sm;	Non-sm;	Sm;	Non-sm;	Sm;	Non-sm;	Sm;	Non-sm;	Sm;	Non-sm;
	3986	16,771	861	4409	287	971	99	343	186	814	130	612	81	278	97	364
Disease severity																
Mild	537 (14)	1728 (10)	29 (3)	116 (3)	60 (21)	169 (17)	19 (19)	33 (10)	27 (15)	87 (11)	23 (18)	89 (15)	20 (25)	37 (13)	9 (9)	53 (15)
Moderately severe	1627 (41)	6355 (38)*	200 (23)	910 (21)	161 (56)	483 (50)	43 (43)	162 (47)*	64 (34)	301 (37)	56 (43)	313 (51)	36 (44)	137 (49)*	40 (41)	120 (33)
Severe	1794 (45)	8484 (51)*	623 (72)	3317 (75)	64 (22)	312 (32)*	35 (35)	141 (41)*	95 (51)	424 (52)	50 (39)	202 (33)	24 (30)	101 (36)*	48 (50)	189 (52)
Extremely severe	28 (1)	204 (1)	9 (1)	66 (2)	2 (1)	7 (1)	2 (2)	7 (2)	0 (0)	2 (0.2)	1 (1)	8 (1)	1 (1)	3 (1)	0 (0)	2 (1)
Abdominal pain	2491 (63)	10,475 (63)	442 (51)	2398 (54)	236 (82)	746 (77)	65 (66)	231 (67)	106 (57)	500 (61)	81 (62)	355 (58)	51 (63)	194 (70)	56 (58)	232 (64)
Watery stool	3643 (91)	15,788 (94)*	851 (99)	4386 (99)*	170 (59)	692 (71)*	91 (92)	324 (95)	171 (92)	780 (96)*	125 (96)	594 (97)	69 (85)	248 (89)	91 (94)	351 (96)
Hospitalization >24 h	720/3839 (19)	3761/16,221 (23)*	260/836 (31)	1502 (35)*	43/278 (16)	189 (20)	25/95 (26)	89 (27)	34/184 (19)	165 (21)	20 (15)	117 (20)	14/80 (2)	63 (23)	16/96 (17)	78 (22)

*p < 0.05 calculated using the chi-square test

Our univariate analysis showed that smokers had a significantly lower severity of diarrhoeal disease than non-smokers (OR: 0.92, 95 % CI: 0.85–0.99, p = 0.035). This significant association remained after adjustment for age, sociodemographic characteristics and the presence of specific pathogens (OR: 0.92, 95 % CI: 0.85–0.99, p = 0.025) (Table 4). However, no dose–response relationship was observed between the number of cigarettes smoked per day and disease severity (Table 5). Moreover, no differences in susceptibility to different antimicrobials were found for either *Shigella* spp. (ampicillin, mecillinam, ciprofloxacin, TMP-SXT and nalidixic acid) or *Vibrio cholerae* (ampicillin, tetracycline, erythromycin, TMP-SXT and ciprofloxacin) (data not shown).

**Table 4:** Univariate and multivariate associations between smoking and severity of diarrhoeal disease

Characteristics	Unadjusted. OR (95 % CI), p	Model 1; OR (95 % CI), p	Model 2; OR (95 % CI), p	Model 3; OR (95 % CI), p	Model 4; OR (95 % CI), p	Model 5; OR (95 % CI) p
Smoking	0.92 (0.85-0.99) 0.035	0.95 (0.88-1.03) 0.239	0.89 (0.83-0.96) 0.004	0.92 (0.85-0.99) 0.025	0.89 (0.83-0.96) 0.004	0.92 (0.85-0.99) 0.025
Age	-					
15–30 years	1	1	1	1	1	1
Above 30 years	0.86 (0.79-0.95) 0.002	0.83 (0.78-0.89) <0.001	0.81 (0.76-0.87) <0.001	0.92 (0.85-0.99) 0.024	0.82 (0.76-0.88) <0.001	0.92 (0.85-0.99) 0.020
Wealth quintile	-	-				
1^st^ (rich)	1	-	1	1	1	1
2^nd^ (upper middle)	1.27 (1.12-1.44) <0.001	-	1.24 (1.11-1.38) <0.001	1.20 (1.08-1.35) 0.001	1.24 (1.11-1.39) <0.001	1.21 (1.08-1.35) 0.001
3^rd^ (middle)	1.60 (1.40-1.83) <0.001	-	1.40 (1.25-1.56) <0.001	1.31 (1.17-1.47) <0.001	1.41 (1.26-1.57) <0.001	1.32 (1.18-1.47) <0.001
4^th^ (lower middle)	2.01 (1.75-2.30) <0.001	-	1.37 (1.22-1.54) <0.001	1.37 (1.22-1.54) <0.001	1.40 (1.25-1.57) <0.001	1.39 (1.24-1.56) <0.001
5^th^ (poor)	1.75 (1.52-2.00) <0.001	-	1.26 (1.12-1.43) <0.001	1.32 (1.67-1.50) <0.001	1.32 (1.17-1.49) <0.001	1.36 (1.20-1.54) <0.001
Illiterate	1.84 (6.12-6.81) <0.001	-	1.41 (1.29-1.52) <0.001	1.40 (1.29-1.51) <0.001	1.43 (1.30-1.52) <0.001	1.39 (1.28-1.51) <0.001
*Vibrio cholerae*	5.61 (4.72-6.65) <0.001	-	-	4.44 (4.05-4.86) <0.001	-	4.28 (3.90-4.69) <0.001
*Shigella* spp.	0.53 (0.45-0.61) <0.001	-	-	-	0.45 (0.40-0.52) <0.001	0.57 (0.50-0.66) <0.001

Only results for male subjects shown. Odds ratios (OR), 95 % confidence intervals (95 % CI) and p values, calculated using ordinal logistic regression, are shown for each model

Dependent variable: disease severity (0 = mild, 1 = moderately severe, 2 = severe, 3 = extremely severe)

Primary exposure: current smoker (0 = no, 1 = Yes)

Adjusting confounder/modifiers:

Model 1: Disease severity, Age (0 = 15–30 years)

Model 2: Model 1 + Wealth index (0 = rich), Illiteracy (0 = literacy)

Model 3: Model 2 + *Vibrio cholerae* (0 = no)

Model 4: Model 2 + *Shigella* spp. (0 = no)

Model 5: Model 1 + all variables

**Table 5:** Dose–response relationship between number of cigarettes smoked per day and severity of diarrhoeal disease (multivariate analysis)

Cigarettes smoked	Unadjusted; OR (95 % CI) p	Adjusted; OR (95 % CI) p
Non-smokers	1.00	1.00
1-9 sticks/day	0.89 (0.82-0.98) 0.024	0.87 (0.79-0.95) 0.005
10-19 sticks/day	0.97 (0.88-1.08) 0.876	0.98 (0.87-1.09) 0.467
≥20 sticks/day	0.88 (0.74-1.06) 0.736	0.93 (0.77-1.13) 0.456

All analyses limited among male; *OR*, Odds ratio; *CI*, Confidence interval; unadjusted and adjusted (models) p value was computed by ordinal logistic regression

Dependent variable: disease severity (0 = mild, 1 = moderately severe, 2 = severe, 3 = extremely severe)

Main exposure: Number of cigarettes smoked (0 = non-smokers, 1 = 1-9 sticks/day, 2 = 10-19 sticks/day, 3 = ≥20 sticks/day)

Adjusting confounder/modifiers: Age (0 = 15-30 years), wealth index (0 = rich), illiteracy (0 = literacy), *Vibrio cholerae* (0 = no), *Shigella* spp. (0 = no)

## Discussion

Despite global tobacco control efforts, tobacco use remains widespread and the burden of smoking-related disease remains a major public health concern [2, 3, 26]. A novel feature of the present study is that we analysed the characteristics of smokers and non-smokers among a large sample of patients who attended a diarrhoeal disease hospital and determined the association between smoking and severity of diarrhoeal disease. The smoking prevalence among participants aged 15 years and above was 19 %, compared with 14 % for the national population [27]. This may be attributed to the specific characteristics of those attending a large, specialised diarrhoeal disease facility in a major urban centre.

Nicotine exposure can result in nausea and diarrhoea related to increased intestinal motor activity [9] and can increase gastric acid secretion, resulting in gastrointestinal conditions such as dyspepsia [28]. While gastric acid secretion is associated with the number of cigarettes smoked a day and duration of smoking history [29], a previous study reported that individuals who smoke more than 20 cigarettes per day had a 1.55 times higher risk of developing non-ulcer dyspepsia [30]. By contrast, the present study found no dose–response relationship between the number of cigarettes smoked daily and diarrhoeal disease severity. This may be because of the small number of individuals in our sample who reported smoking more than 20 cigarettes per day.

Smokers were more likely to be infected with *Shigella* spp. and less likely to develop *Vibrio cholerae* infections when compared with non-smokers. Given that production of hydrochloric acid in the stomach may be elevated in smokers, larger numbers of ingested *Vibrio cholerae* may not survive and thus may require higher infective dose (>10^5^ organisms). By contrast, the infective dose of *Shigella* spp. to allow it to pass through the gastric acid barrier is as low as 10 organisms. Additionally, smoking compromises leukocyte function (including neutrophils, monocytes, T and B cells), thereby and increasing the risk of infection [31]. This effect may also increase the risk of shigellosis infection via mucosal membranes because of decreased humoral immune responses [32].

Smoking among women is highly stigmatised in Bangladesh for cultural reasons, with the exception of a few tribal groups [33]. This explains the fact that 97 % of smokers in our sample were male. More than two thirds of smokers were in the three lowest wealth quintiles. Low earnings compounded by social disadvantage are strong determinants of mental illness such as depression, which in turn leads to adverse health behaviours such as smoking uptake in adolescence. Although controversial, previous work suggests that smokers may exploit the calming effects of nicotine to reduce anxiety [34, 35]. Moreover, illiteracy and lack of access to electronic media may lead to lower awareness of the long term health consequences of smoking.

The proportion of subjects who habitually boiled their drinking water was lower among smokers than non-smokers in univariate analysis. Although this is an important predictor of diarrhoea, it can also be considered a proxy indicator for socioeconomic status. This result is therefore expected given that smokers are more likely to be socioeconomically disadvantaged. Additionally, more than half of the participants had received antimicrobials prior to coming to hospital, which may be a concern given the potential for antimicrobial resistance to develop. However, we found that most commercially available antimicrobials such as ciprofloxacin (96 %), mecillinam (95 %) and ceftriaxone (100 %) were effective against *Shigella* spp., and 100 % of *Vibrio cholerae* isolates showed susceptibility to ciprofloxacin (data not shown).

Significant differences in disease severity were found between smokers and non-smokers presenting with diarrhoeal illnesses. These observations may be correlated without a casual pathway because of the common symptom profiles associated with different enteric pathogens. While frequent consumption of contaminated food and water may be the underlying cause of diarrhoeal disease in our sample, differences in disease severity may have also been a result of health behaviours such as calorie restriction, which is more widespread among middle-aged individuals. We were unable to evaluate these possible effects, however, because of a lack of data.

### Study limitations

Given that the present study was conducted among patients presenting with diarrhoeal disease attending a specialised facility in an urban area, our results may not be generalizable to the wider national population. There was also a lack of retrospective data on lifetime smoking behaviour which may have caused the effect of smoking on disease severity to be underestimated. Furthermore, lack of data prevented us from adjusting for the presence of functional gastrointestinal disorders. Another concern was the lack of data on the duration of vomiting when determining disease severity scores. However, the study’s strengths included the large data sample employed, the low probability of bias of our systematic sampling methods and the high laboratory performance for detecting specific causative pathogens.

## Conclusions

The distinct clinical and etiological patterns of diarrhoeal disease observed in the present study suggest that smokers in our sample differed from non-smokers. Despite the limitations of the present study, our results contribute significantly to the evidence base on the relationship between smoking and diarrhoea and its public health implications. Greater priority should be given by policy makers and public health practitioners to implement tobacco control programmes and restricting access to tobacco among the general population, especially among teenagers and young adults, to reduce the disease burden caused by both active and passive smoking. Moreover, further in-depth research is needed to explore the impact of smoking on diarrhoeal illnesses of diverse aetiology.

## Abbreviations

95 % CI: 95 % confidence intervals
DDSS: Diarrhoeal Disease Surveillance System
ICDDR,B: International Centre for Diarrhoeal Disease Research, Bangladesh
OR: Odds ratio
TMP-STX: Trimethoprim-sulfamethoxazole
